# Alpinetin Attenuates Persistent Inflammation, Immune Suppression, and Catabolism Syndrome in a Septic Mouse Model

**DOI:** 10.1155/2021/9998517

**Published:** 2021-07-05

**Authors:** Yukun Liu, Kang Wang, Qaunrui Feng, Yongsheng Zhang, Chuntao Wang, Qinxin Liu, Xinghua Liu, Xiang Wang, Wei Gao, Xiangjun Bai, Zhanfei Li, Yuchang Wang

**Affiliations:** ^1^Department of Plastic Surgery, Tongji Hospital of Tongji Medical College, Huazhong University of Science and Technology, Wuhan 430030, China; ^2^Digestive Disease Center, Seventh Affiliated Hospital, Sun Yat-sen University, Shenzhen 518107, China; ^3^Trauma Center/Department of Emergency and Traumatic Surgery, Tongji Hospital of Tongji Medical College, Huazhong University of Science and Technology, Wuhan 430030, China

## Abstract

Patients who survive the acute phase of sepsis can progress to persistent inflammation, immunosuppression, and catabolism syndrome (PICS), which usually results in extended recovery periods and multiple complications. Alpinetin is a flavonoid isolated from Alpinia katsumadai Hayata that has been demonstrated to have anti-inflammatory, antibacterial, and antioxidant activities. The aim of this study was to investigate whether the administration of alpinetin could attenuate PICS in a septic mouse model. Mice were randomly divided into four groups: the (1) sham-operated group, (2) sham+alpinetin (1 mg/kg intravenously infused for once per day after sham operation), (3) cecal ligation and puncture (CLP), and (4) CLP+alpinetin (50 mg/kg intravenously infused for once per day after CLP). Eight days after sham operation or CLP surgery, mice were euthanized for subsequent examination. Alpinetin significantly improved the survival of septic mice. Also, it attenuated the CLP-induced persistent inflammation, immunosuppression, and catabolism syndrome. The level of plasma proinflammatory cytokines and apoptosis of T lymphocytes were obviously decreased by alpinetin as well. Moreover, oxidative stress in the organs was compelling lower in the alpinetin-treated CLP mice. In this clinically relevant model of sepsis, alpinetin ameliorates CLP-induced organ dysfunction and improves the likelihood of survival, possibly through suppressing the inflammatory response, oxidative stress, and apoptosis. These findings suggested that alpinetin could be a potential novel therapeutic approach to prevent sepsis-induced PICS.

## 1. Introduction

The advancements of diagnosis and management have substantially improved the overall survival rate of sepsis in the past few decades. Unfortunately, long-term outcomes in sepsis survivors have not been improved over time, usually resulting in a state of chronic critical illness [1]. Some sepsis survivors present with typical syndromes that follow the SIRS-CARS reaction: persistent inflammation, immunosuppression, and catabolism syndrome (PICS), marked by recurrent nosocomial infections, inadequate nutrition, and the need for long-term skilled nursing. Physiologically, these states are generally characterized by sustained inflammation, suppressed host immunity, and the loss of lean muscle mass [2, 3]. Many patients are suffering from prolonged recovery times and often progress to chronic critical illness (CCI) with less than 50% of patients surviving 1 year after hospital discharge [4, 5]. Nevertheless, no specific and effective pharmacological intervention is currently available for PICS.

Although the PICS hypothesis has been validated early in 2012 [6], the precise mechanism remains as yet incomplete. Unbalance of persistent inflammation and progressive immunosuppression were widely accepted to be the basic mechanisms of PICS. This persistent inflammation is characterized by increased concentration of plasma IL-6, neutrophilia, etc. The reason contributing to an immunosuppressive state is marked by T cell exhaustion and increased proportions of regulatory T cells (Tregs), lymphopenia, etc. [7]. Lymphopenia is considered a typical sign of immunosuppression in clinical practice [6]. Therefore, the development of new agents that have the ability of anti-inflammation in addition to modulating immunity may be useful for the treatment of PICS.

Alpinetin is a Chinese traditional medicine usually isolated from the plants of Alpinia katsumadai Hayata. This drug has been reported to have varieties of biological activities, including antibacterial, antioxidative, and anti-inflammatory [8, 9]. Numerous studies have suggested that alpinetin has a protective effect on ulcerative colitis and kidney injury in mice by regulating inflammation [8, 9]. However, whether alpinetin had protective effects against sepsis-induced PICS has not yet been fully explored. In the present study, we aimed to testify the protective effects of alpinetin on PICS.

## 2. Methods

### 2.1. Animal

Six-week-old male C57BL/6 mice were housed in standard environmental conditions with a 12 h/12 h light/dark conditions and fed with standard commercial chow with free access to water. This study was approved by the local institutional review board, and the experiments were performed and were approved by the Animal Ethics Committee of Tongji Hospital, Tongji Medical College, Huazhong University of Science and Technology.

### 2.2. PICS Model of Sepsis

Sepsis-induced PICS models were established by CLP as previously described [3]. Briefly, seven- to eight-week-old male mice (22–26 g) were used in all experiments. Mice were anesthetized with pentobarbital sodium (40 mg/kg) injected intraperitoneally (i.p.). Sham-operated mice underwent the same protocol without the CLP procedure. Finally, mice were resuscitated with sterile saline (50 mL/kg) injected subcutaneously.

### 2.3. Experimental Protocol

C57BL/6 male mice were randomly divided into four groups: the (1) sham-operated group, (2) sham +alpinetin group, (3) CLP group, and (4) CLP+alpinetin (50 mg/kg) group. Eight days after sham operation or CLP surgery, mice were euthanized for subsequent examination. Alpinetin was purchased from the National Institute for the Control of Pharmaceutical and Biological Products (Beijing, China). The dose of alpinetin was selected according to previous reports [10].

### 2.4. Flow Cytometry

Single-cell suspensions of the spleen were prepared as previously described. Cell counts were determined using a Coulter Ac T 10 cell counter (Beckman Coulter, Brea, CA, USA). Cells were then resuspended in a flow cytometry buffer (1% bovine serum PBS), and nonspecific binding was blocked by a preincubation with 5% rat serum (Invitrogen, Life Technologies, Grand Island, NY, USA) and 1 *μ*L/sample of Fc Block (BD Pharmingen, San Jose, CA, USA). The following cell types were identified using the following antibody combinations: neutrophils (Ly-6G and CD11b) and CD4 T cells, CD3 and CD4 T cells, and CD8 T cells (CD3 and CD8).

### 2.5. ELISA

TNF-*α* and IL-6 levels in serum were measured using ELISA kits (BioLegend, CA, USA) according to the manufacturer's instructions.

### 2.6. Histological Examination

Lung tissues were fixed overnight in 4% paraformaldehyde and embedded in paraffin. Then, sections were cut into 5 *μ*m, and hematoxylin-eosin (H&E) staining was performed. Histopathological examinations were carried out using a microscope (RX51, Olympus Optical Co., Ltd., Tokyo, Japan). The histological examination was performed in a blinded fashion using a scoring system previously validated and described.

### 2.7. Measurement of ROS and SOD Level

Tissues of the lung and spleen were cut into cubes, and the dispersed cells filtered with a 300-mesh nylon net. After washing with cold PBS, the fluorescence intensity of ROS was measured with excitation wavelength at 500 nm and emission wavelength at 525 nm using reactive oxygen species assay kits (Nanjing Jiancheng Bioengineering Institute, China) following the manufacturer's protocols.

Tissues of the lung and spleen were homogenized using a tissue grinder and determined using BCA Protein Assay Kits. The activity of SOD was assessed using SOD assay kits (Nanjing Jiancheng Bioengineering Institute) according to the manufacturer's instructions.

### 2.8. Statistical Analysis

All statistical analyses were completed using GraphPad Prism 8.0 (USA). All data were presented as mean ± SD. Normally distributed data were determined by one-way analysis of variance (ANOVA), followed by the Tukey post hoc test. Nonnormally distributed data were analyzed with nonparametric Wilcoxon tests. Survival data were analyzed using the Kaplan-Meier method, and survival curves were compared using the log-rank test and Gehan-Breslow-Wilcoxon test in univariate analysis. Statistical significance was defined as *p* value < 0.05.

## 3. Results

### 3.1. Alpinetin Improved the Survival of CLP Septic Mice

**Figure 1:**
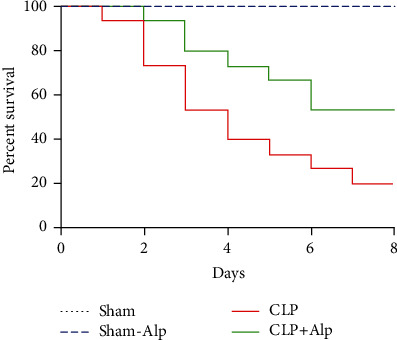
Effect of alpinetin on the survival of CLP septic mice. Mice were intravenously administered with alpinetin (50 mg/kg) in the sham+alpinetin and CLP+alpinetin groups, and the survival rates of the mice were monitored for 8 days. Survival data were analyzed using the Kaplan-Meier method, and survival curves were compared using the log-rank test and Gehan-Breslow-Wilcoxon test in univariate analysis. ^∗∗∗^*p* < 0.001, CLP vs. sham; ^#^*p* < 0.05, CLP-Alp vs. CLP.

To evaluate the effect of alpinetin administration on the survival of CLP-operated mice, mice were divided into four groups as shown in Figure 1 and monitored for 8 days. The survival rate in the CLP group (survival: 6 of 30 mice, 20.0%) was significantly lower than that in the sham and sham+alpinetin groups (survival: 10 of 10 mice, 100% in sham and sham+alpinetin groups, *p* < 0.001). Interestingly, the administration of alpinetin significantly improved the survival rate to 53.3% in the CLP+alpinetin group (survival: 8 of 15 mice, *p* < 0.05).

### 3.2. Alpinetin Alleviated Inflammation in CLP-Induced PICS in Mice

**Figure 2:**
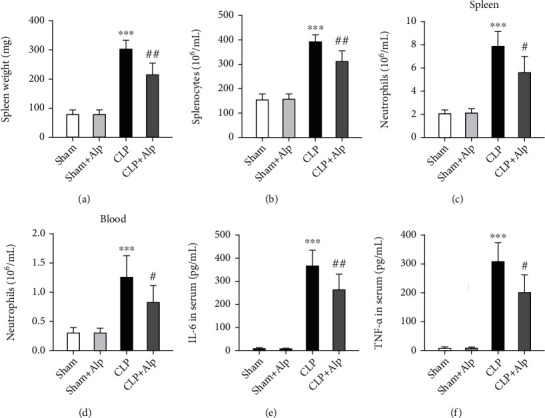
Effects of alpinetin on splenic myelopoiesis and leukocytosis eight days after the procedure. The spleen and blood were collected and analyzed eight days after the cecal ligation and puncture (CLP) operation. (a) Weight, (b) total number of WBCs, (c) neutrophils in the spleen, and (d) neutrophils in the blood. (e) IL-6 and (f) TNF-*α* in serum were quantified by using ELISA kits. Sham (*n* = 10), sham+Alp (*n* = 10), CLP (*n* = 6), and CLP+Alp (*n* = 8). Data are shown as mean ± SD. ^∗∗∗^*p* < 0.001, CLP vs. sham; ^#^*p* < 0.05, ^##^*p* < 0.01, CLP-Alp vs. CLP.

To determine the effects of alpinetin on inflammation, numbers of neutrophils in the spleen and blood were detected eight days after CLP. Weight of the spleen, splenocytes, and number of neutrophils in the spleen and blood increased in the CLP group, which were consistent with the characteristics of PICS. Alpinetin significantly decreased the weight of the spleen and the number of white blood cells compared with those in the CLP group (Figures 2(a) and 2(b)). Additionally, we observed that the number of neutrophils in the spleen and blood was significantly decreased in the CLP+alpinetin group (Figures 2(c) and 2(d)). Furthermore, there were notable increases in IL-6 and TNF-*α* levels in the serum of CLP which were reduced by alpinetin treatment (Figures 2(e) and 2(f)). These results suggested that alpinetin significantly attenuated inflammation in sepsis-induced PICS.

### 3.3. Alpinetin Improved Immunosuppression in CLP-Induced PICS

**Figure 3:**
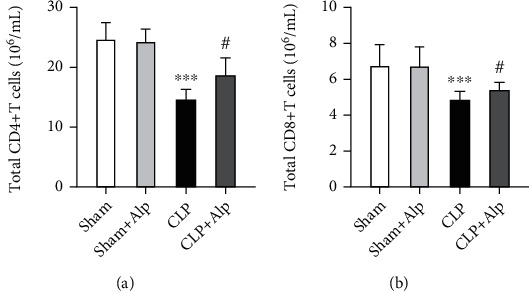
Effects of alpinetin on splenic T cells eight days after the procedure. After eight days, animals were sacrificed and the spleens were removed. Flow cytometry was then used to characterize the number of (a) total CD4^+^ T cells and (b) total CD8^+^ T cells in each group. Sham (*n* = 10), sham+Alp (*n* = 10), CLP (*n* = 6), and CLP+Alp (*n* = 8). Data are shown as mean ± SD. ^∗∗∗^*p* < 0.001, CLP vs. sham; ^#^*p* < 0.05, CLP-Alp vs. CLP.

Lymphopenia (lymphocyte count < 800/mm^3^) is considered to be an important clinical feature in PICS [6]. The loss of T cells is a key determinant of immune suppression. This is of particular importance as studies did reveal that persistent lymphopenia is related to increased mortality and secondary infections in severely ill intensive care patients [11]. In a murine PICS model, lymphopenia was depicted in a quantitative loss of CD4^+^ and CD8^+^ T cells [3]. In our study, we examined splenic T cell numbers eight days after CLP. We observed that the total CD4^+^ and CD8^+^ T cell numbers from septic mice are significantly decreased in the CLP group and significantly suppressed in the CLP+alpinetin group (Figures 3(a) and 3(b)), suggesting that alpinetin improved the immunosuppression in CLP-induced PICS.

### 3.4. Alpinetin Improved Catabolism in CLP-Induced PICS

**Figure 4:**
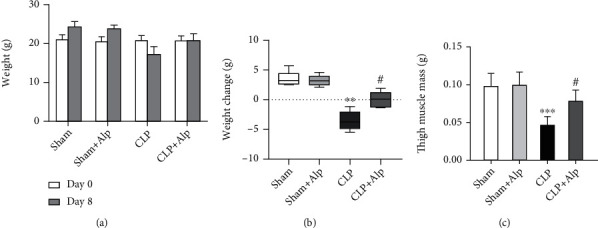
Effects of alpinetin on weight changes after the procedure. (a) Mice were weighed before the operation (day 0) and on the eighth day after the procedure (day 8). Groups were then compared in terms of (b) absolute change in weight and (c) thigh muscle weight. Sham (*n* = 10), sham+Alp (*n* = 10), CLP (*n* = 6), and CLP+Alp (*n* = 8). Data are shown as mean ± SD. ^∗∗^*p* < 0.01, ^∗∗∗^*p* < 0.001, CLP vs. sham; ^#^*p* < 0.05, CLP-Alp vs. CLP.

Most septic patients display a rapid loss of lean muscle mass due to sustained catabolism. The decrease in muscle mass associated with sepsis is not due to a decline in protein synthesis, but rather attributed to an increase in protein breakdown [12]. Literature has implicated an ongoing inflammatory response as the driving force behind prolonged catabolism in sepsis [13]. In a murine PICS model, mice had a leg muscle mass loss of approximately 50% eight days after CLP [3]. To validate this, we evaluated the weight change and thigh muscle mass of mice. In our study, both weight and thigh muscle mass were significantly decreased in the CLP group, but largely increased in the CLP+alpinetin group (Figures 4(a) and 4(b)), suggesting that alpinetin may help to improve nutritional status in PICS.

### 3.5. Alpinetin Treatment Inhibited Apoptosis of CD4^+^ and CD8^+^ T Cells

**Figure 5:**
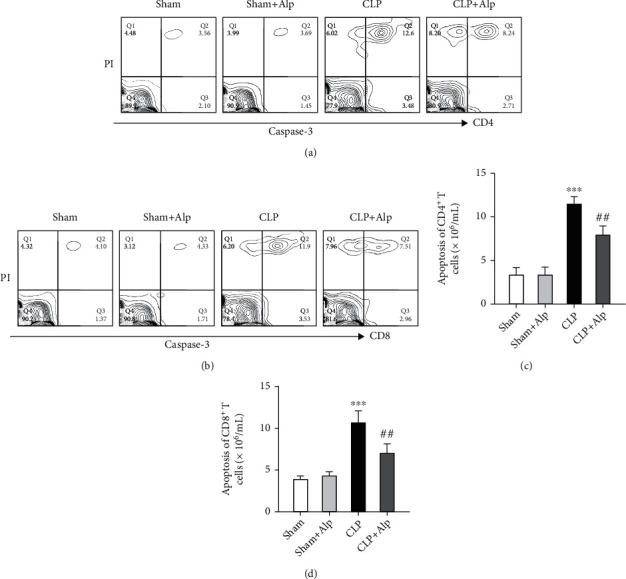
Effects of alpinetin on apoptosis of CD4^+^ and CD8^+^ T cells in the spleen. Flow cytometry was then used to evaluate the apoptosis by detecting caspase-3 activation and cell death (PI positive) of CD4^+^ (a, c) and CD8^+^ (b, d) T cells in the spleen on the eighth day. Sham (*n* = 8), sham+Alp (*n* = 8), CLP (*n* = 6), and CLP+Alp (*n* = 8). Data are shown as mean ± SD. ^∗∗∗^*p* < 0.001, CLP vs. sham; ^##^*p* < 0.01, CLP-Alp vs. CLP.

Lymphocyte apoptosis has been reported to play an important role in the pathogenesis of sepsis and is thought to be the main reason that contributes to lymphopenia [14]. To further confirm the effects of alpinetin on T lymphocyte apoptosis, flow cytometry was used to detect the apoptosis of CD4^+^ and CD8^+^ T lymphocytes in vivo and apoptosis was labeled with caspase-3^+^PI^+^. The percentages of CD4^**+**^ and CD8^**+**^ T lymphocyte apoptosis were significantly increased in the CLP group, which was in accordance with the previous study. Interestingly, alpinetin administration significantly suppressed the apoptosis of CD4^+^ and CD8^+^ T lymphocytes (Figures 5(a) and 5(b)). In a word, our results demonstrated that alpinetin inhibited the activation of caspase-3-dependent apoptosis.

### 3.6. Alpinetin Reduces Lung Injury and MPO Activity

**Figure 6:**
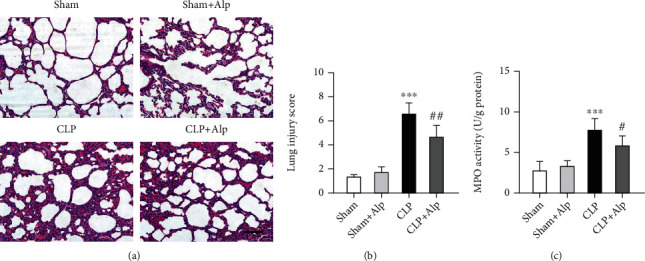
Effects of alpinetin on lung injury and MPO activity. (a) Representative images of H&E-stained lung tissues from different groups. (b) Lung injury score. (c) MPO activity was analyzed using MPO Assay Kits. The scale bar is 50 *μ*m. Sham (*n* = 8), sham+Alp (*n* = 8), CLP (*n* = 6), and CLP+Alp (*n* = 8). Data are shown as mean ± SD. ^∗∗∗^*p* < 0.001, CLP vs. sham; ^##^*p* < 0.01, CLP-Alp vs. CLP.

Sustained tissue damage and multiple organ failure are another characteristic of PICS. It is commonly accepted that the lung is the primary target of organ damage in sepsis. Thus, to further investigate the effect of alpinetin on lung inflammation, lung tissues were collected for cell H&E staining and MPO activity. Hemorrhage, alveolar septal thickening, and leukocyte infiltration were observed in PICS mice compared with the sham group. However, these histopathological changes and the lung injury score were significantly attenuated by alpinetin treatment (Figures 6(a) and 6(b)). Inflammatory cell infiltration plays a critical role in lung inflammation and is overwhelmingly the highest contributor to tissue damage. Additionally, myeloperoxidase (MPO) activity is considered to be a marker of inflammatory cell infiltration in lung tissues [15]. As shown in Figure 6(c), MPO activity significantly increased in lung tissues in PICS mice. However, MPO activity was dramatically reduced by the administration of alpinetin. These findings suggest that alpinetin has protective effects against inflammatory injury in PICS.

### 3.7. Alpinetin Ameliorates Oxidative Stress in Septic Mice

**Figure 7:**
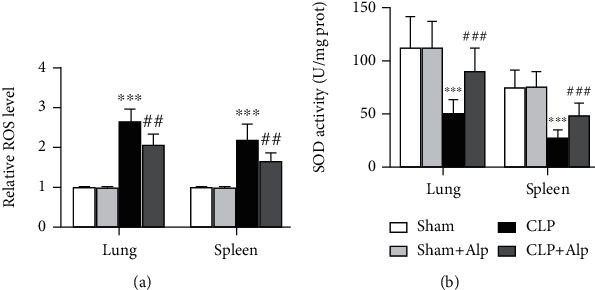
Effects of alpinetin on oxidative stress in CLP-induced PICS. After eight days, animals were sacrificed and the lung, liver, and kidney were removed. (a) The ROS levels in the mouse lung and spleen were assessed using commercial reactive oxygen species assay kits. (b) The SOD activity in mouse lung and spleen tissues was evaluated using commercial SOD assay kits. Sham (*n* = 8), sham+Alp (*n* = 8), CLP (*n* = 6), and CLP+Alp (*n* = 6). Data are shown as mean ± SD. ^∗∗∗^*p* < 0.001, CLP vs. sham; ^##^*p* < 0.01, ^###^*p* < 0.001, CLP-Alp vs. CLP.

Sepsis is characterized by an excess of reactive oxygen species, inducing cellular damage and death, depletion of antioxidants, and accumulation of markers of oxidative stress [16]. Alleviating oxidative stress is considered one of the important mechanisms to treat lung injury [17, 18]. The level of oxidative stress was analyzed by determining ROS level and SOD activity in the lung and spleen. We observed an increased ROS in the lung and spleen in the PICS group, and alpinetin reverts back the level of these organs (Figure 7(b)). Regarding antioxidant enzymes, the SOD activity showed significant decrease after sepsis in the meanwhile, and alpinetin was effective in increasing SOD activity level in the lung and spleen (Figure 7(b)).

## 4. Discussion

In this study, we used a rational and scientific method to demonstrate that the CLP procedure evoked PICS, characterized by elevated neutrophil number, increased plasma levels of IL-6 and TNF-*α*, lymphopenia, and loss of weight, which were improved by alpinetin in a mouse model of sepsis-induced PICS. As a result, the intravenous administration of alpinetin improved the survival of the CLP septic mice. Furthermore, alpinetin significantly reduced the apoptosis of CD4^+^ and CD8^+^ T lymphocyte and lung injury and reduced lung and spleen superoxide production in PICS mice. Accordingly, these findings suggest that alpinetin attenuates CLP-induced PICS and may be useful as a therapeutic agent to relieve related organ injury.

Alpinetin, a novel plant flavonoid isolated from Alpinia katsumadai Hayata, has anti-inflammatory and antioxidative effects on variable disease processes, such as ulcerative colitis, acute kidney injury, and endometritis [8–10, 19]. In the mouse model of LPS-induced liver injury, alpinetin inhibited liver injury through reducing inflammatory cell infiltration and proinflammatory cytokine release [9]. In an in vivo model of ulcerative colitis, dextran sulfate sodium-induced intestinal barrier dysfunction, inflammation, and oxidative stress were attenuated or prevented by alpinetin [8].

The CLP model has been widely used as the gold standard in sepsis models, as it closely mimics the clinical condition of human sepsis [20]. The previous study has implicated that mice which survived eight days after CLP displayed PICS characteristics including lymphocyte depletion, circulating myeloid cell increase, and weight loss [3]. In our present study, results showed that mice that survived eight days not only displayed PICS characteristic but also accompanied by lung injury.

Recent studies demonstrated that the increasing apoptosis of immune cells plays a pivotal role in immunosuppression and consequent organ dysfunction, which were observed in sepsis [14, 21]. Animal studies showed that the blockade of cell apoptosis could improve the survival rate in a sepsis model [14]. Studies have revealed that alpinetin could attenuate chronic obstructive pulmonary disease (COPD) and colitis by inhibiting apoptosis [22, 23]. Su et al. have recently reported that alpinetin can reduce the activity of caspase-3 and caspase-9 in COPD rats, inhibit the occurrence of alveolar cell apoptosis, and reduce the release of inflammatory factors by reducing the activities of TGF-*β*1, TNF-*α*, and *α*-SMA [23]. In the present study, there was obvious downregulation of the expression of caspase-3 in T cells of PICS mice after alpinetin intervention. Alpinetin ameliorated lymphopenia and the apoptosis of CD4^+^ T and CD8^+^ T lymphocytes in the spleen of PICS mice. Results indicated that alpinetin could attenuate cell death and immunosuppression of T lymphocytes induced by sepsis. Additionally, apoptosis can be triggered by a variety of stimuli including inflammatory cytokines and ROS. ROS has been extensively implicated in T cell hyporesponsiveness, apoptosis, and activation [24, 25]. To validate this hypothesis, the levels of ROS and SOD were measured to assess the degree of oxidative stress in the spleen of PICS mice. In our study, PICS triggered ROS overproduction and SOD hypoactivity in the spleen, indicating that the elevated levels of oxidative stress exist and may facilitate T lymphocyte apoptosis in PICS mice. However, both apoptosis of T cells and oxidative stress in the spleen were suppressed by alpinetin. Therefore, alpinetin may exert its antiapoptotic effect by reducing the production of superoxide. Taken together, these results indicate that alpinetin attenuates CLP-induced PICS through its counterregulatory action to mediate the resolution of inflammation, oxidative stress, and apoptosis.

Death from sepsis is not thought to be related to infection itself but associated with organ failure that results from the systemic response to infection [26]. Lung injury is one of the most frequent organ dysfunctions in sepsis. We found that alpinetin administration exhibited the tendency to ameliorate lung injury as shown by decreased inflammatory cell infiltration and MPO. Evidence has indicated that oxidative stress, which is induced by ROS accumulation and reduced activity of antioxidant enzymes, plays a crucial role in the progress of organ failure in sepsis [16, 18, 27–29]. In our study, along with the increase in ROS, the activity of SOD decreases in the lung of PICS mice. However, alpinetin exhibited antioxidative action in the lung of PICS as evidenced by upregulation of SOD, accompanied by the decrease in ROS. These findings indicated that alpinetin may attenuate inflammation and lung injury via inhibiting oxidative stress in the lung of PICS.

## 5. Conclusions

In conclusion, the present study described a novel function of alpinetin in alleviating CLP-induced PICS. Alpinetin attenuates PICS and related organ injury in the mouse model by anti-inflammation effect in addition to inhibiting apoptosis of T lymphocytes, which may be involved in oxidative stress. Further studies, in particular clinical trials, are necessary to verify the potential adjuvant effect of alpinetin in PICS.

## Data Availability

The data are presented within the paper. Additional raw data are available on request from the corresponding author.

## References

[1] Shankar-Hari M., Rubenfeld G. D. (2016). Understanding long-term outcomes following sepsis: implications and challenges. *Current Infectious Disease Reports*.

[2] Horiguchi H., Loftus T. J., Hawkins R. B. (2018). Innate immunity in the persistent inflammation, immunosuppression, and catabolism syndrome and its implications for therapy. *Front Immunol.*.

[3] Pugh A. M., Auteri N. J., Goetzman H. S., Caldwell C. C., Nomellini V. (2017). A murine model of persistent inflammation, immune suppression, and catabolism syndrome. *International Journal of Molecular Sciences*.

[4] Hawkins R. B., Raymond S. L., Stortz J. A. (2018). Chronic critical illness and the persistent inflammation, immunosuppression, and catabolism syndrome. *Frontiers in Immunology*.

[5] Mira J. C., Gentile L. F., Mathias B. J. (2017). Sepsis pathophysiology, chronic critical illness, and persistent inflammation-immunosuppression and catabolism syndrome. *Critical Care Medicine*.

[6] Gentile L. F., Cuenca A. G., Efron P. A. (2012). Persistent inflammation and immunosuppression: a common syndrome and new horizon for surgical intensive care. *Journal of Trauma and Acute Care Surgery*.

[7] Bergmann C. B., Beckmann N., Salyer C. E., Crisologo P. A., Nomellini V., Caldwell C. C. (2020). Lymphocyte immunosuppression and dysfunction contributing to persistent inflammation, immunosuppression and catabolism syndrome (PICS). *Shock*.

[8] Tan Y., Zheng C. (2018). Effects of alpinetin on intestinal barrier function, inflammation and oxidative stress in dextran sulfate sodium-induced ulcerative colitis mice. *The American Journal of the Medical Sciences*.

[9] Liu T. G., Sha K. H., Zhang L. G., Liu X. X., Yang F., Cheng J. Y. (2019). Protective effects of alpinetin on lipopolysaccharide/d-galactosamine-induced liver injury through inhibiting inflammatory and oxidative responses. *Microbial Pathogenesis*.

[10] Huang Y., Zhou L. S., Yan L., Ren J., Zhou D. X., Li S. S. (2015). Alpinetin inhibits lipopolysaccharide-induced acute kidney injury in mice. *International Immunopharmacology*.

[11] Adrie C., Lugosi M., Sonneville R. (2017). Persistent lymphopenia is a risk factor for ICU-acquired infections and for death in ICU patients with sustained hypotension at admission. *Annals of Intensive Care*.

[12] Klaude M., Mori M., Tjader I., Gustafsson T., Wernerman J., Rooyackers O. (2012). Protein metabolism and gene expression in skeletal muscle of critically ill patients with sepsis. *Clinical Science (London, England)*.

[13] Rosenthal M., Gabrielli A., Moore F. (2016). The evolution of nutritional support in long term ICU patients: from multisystem organ failure to persistent inflammation immunosuppression catabolism syndrome. *Minerva Anestesiologica*.

[14] Luan Y. Y., Yao Y. M., Xiao X. Z., Sheng Z. Y. (2015). Insights into the apoptotic death of immune cells in sepsis. *Journal of Interferon & Cytokine Research*.

[15] Wang Y. C., Liu Q. X., Zheng Q. (2019). Dihydromyricetin alleviates sepsis-induced acute lung injury through inhibiting NLRP3 inflammasome-dependent pyroptosis in mice model. *Inflammation*.

[16] Mantzarlis K., Tsolaki V., Zakynthinos E. (2017). Role of oxidative stress and mitochondrial dysfunction in sepsis and potential therapies. *Oxidative Medicine and Cellular Longevity*.

[17] Ni Y. L., Shen H. T., Su C. H. (2019). Nerolidol suppresses the inflammatory response during lipopolysaccharide-induced acute lung injury via the modulation of antioxidant enzymes and the AMPK/Nrf-2/HO-1 pathway. *Oxidative Medicine and Cellular Longevity*.

[18] Xia W., Pan Z., Zhang H., Zhou Q., Liu Y. (2020). Inhibition of ERR*α* aggravates sepsis-induced acute lung injury in rats via provoking inflammation and oxidative stress. *Oxidative Medicine and Cellular Longevity*.

[19] Liang Y., Shen T., Ming Q. (2018). Alpinetin ameliorates inflammatory response in LPS-induced endometritis in mice. *International Immunopharmacology*.

[20] Dejager L., Pinheiro I., Dejonckheere E., Libert C. (2011). Cecal ligation and puncture: the gold standard model for polymicrobial sepsis?. *Trends in Microbiology*.

[21] Aziz M., Jacob A., Wang P. (2014). Revisiting caspases in sepsis. *Cell Death & Disease*.

[22] Miao Y., Lv Q., Qiao S. (2019). Alpinetin improves intestinal barrier homeostasis via regulating AhR/suv39h1/TSC2/mTORC1/autophagy pathway. *Toxicology and Applied Pharmacology*.

[23] Su Y., Tao X., Xu J. (2020). Protective effect of alpinetin on rats with chronic obstructive pulmonary disease. *Food Science & Nutrition*.

[24] Belikov A. V., Schraven B., Simeoni L. (2015). T cells and reactive oxygen species. *Journal of Biomedical Science*.

[25] Signorile A., Ferretta A., Ruggieri M. (2020). Mitochondria, oxidative stress, cAMP signalling and apoptosis: a crossroads in lymphocytes of multiple sclerosis, a possible role of nutraceutics. *Antioxidants*.

[26] Singer M., Deutschman C. S., Seymour C. W. (2016). The Third International Consensus Definitions for Sepsis and Septic Shock (Sepsis-3). *Journal of the American Medical Association*.

[27] Minter B. E., Lowes D. A., Webster N. R., Galley H. F. (2020). Differential effects of mitoVitE, *α*-tocopherol and trolox on oxidative stress, mitochondrial function and inflammatory signalling pathways in endothelial cells cultured under conditions mimicking sepsis. *Antioxidants*.

[28] Kim J. Y., Leem J., Hong H. L. (2020). Protective effects of SPA0355, a thiourea analogue, against lipopolysaccharide-induced acute kidney injury in mice. *Antioxidants*.

[29] Zhong X., He J., Zhang X. (2019). UCP2 alleviates tubular epithelial cell apoptosis in lipopolysaccharide-induced acute kidney injury by decreasing ROS production. *Biomedicine & Pharmacotherapy*.

